# Filarial Worms Reduce *Plasmodium* Infectivity in Mosquitoes

**DOI:** 10.1371/journal.pntd.0000963

**Published:** 2011-02-08

**Authors:** Matthew T. Aliota, Cheng-Chen Chen, Henry Dagoro, Jeremy F. Fuchs, Bruce M. Christensen

**Affiliations:** 1 Department of Pathobiological Sciences, University of Wisconsin-Madison, Madison, Wisconsin, United States of America; 2 Department of Microbiology and Immunology, National Yang-Ming University, Taipei, Taiwan; 3 Papua New Guinea Institute of Medical Research, Madang, Papua New Guinea; Johns Hopkins Bloomberg School of Public Health, United States of America

## Abstract

**Background:**

Co-occurrence of malaria and filarial worm parasites has been reported, but little is known about the interaction between filarial worm and malaria parasites with the same *Anopheles* vector. Herein, we present data evaluating the interaction between *Wuchereria bancrofti* and *Anopheles punctulatus* in Papua New Guinea (PNG). Our field studies in PNG demonstrated that *An. punctulatus* utilizes the melanization immune response as a natural mechanism of filarial worm resistance against invading *W. bancrofti* microfilariae. We then conducted laboratory studies utilizing the mosquitoes *Armigeres subalbatus* and *Aedes aegypti* and the parasites *Brugia malayi*, *Brugia pahangi, Dirofilaria immitis*, and *Plasmodium gallinaceum* to evaluate the hypothesis that immune activation and/or development by filarial worms negatively impact *Plasmodium* development in co-infected mosquitoes. *Ar. subalbatus* used in this study are natural vectors of *P. gallinaceum* and *B. pahangi* and they are naturally refractory to *B. malayi* (melanization-based refractoriness).

**Methodology/Principal Findings:**

Mosquitoes were dissected and *Plasmodium* development was analyzed six days after blood feeding on either *P. gallinaceum* alone or after taking a bloodmeal containing both *P. gallinaceum* and *B. malayi* or a bloodmeal containing both *P. gallinaceum* and *B. pahangi*. There was a significant reduction in the prevalence and mean intensity of *Plasmodium* infections in two species of mosquito that had dual infections as compared to those mosquitoes that were infected with *Plasmodium* alone, and was independent of whether the mosquito had a melanization immune response to the filarial worm or not. However, there was no reduction in *Plasmodium* development when filarial worms were present in the bloodmeal (*D. immitis*) but midgut penetration was absent, suggesting that factors associated with penetration of the midgut by filarial worms likely are responsible for the observed reduction in malaria parasite infections.

**Conclusions/Significance:**

These results could have an impact on vector infection and transmission dynamics in areas where *Anopheles* transmit both parasites, i.e., the elimination of filarial worms in a co-endemic locale could enhance malaria transmission.

## Author Summary

The parasites that cause malaria and human lymphatic filariasis are both transmitted by mosquitoes, and often times in areas where these two diseases are co-endemic, mosquitoes in the genus *Anopheles* transmit both parasites. Currently, it is unknown how parasite transmission is effected when malaria and filarial worm parasites share the same vector. Here, we show that when these two parasites share the same mosquito host, there is a significant reduction in the intensity and prevalence of *Plasmodium* infections. This reduction occurs regardless of the mosquito having a melanization-based immune response activated by filarial worms or when filarial worms successfully develop within the mosquito host. We also observed that filarial worm penetration of the mosquito midgut was necessary for malaria parasite reduction to occur. Our study provides new insight into the relationship between malaria and filarial worm parasites with their mosquito host, which could impact transmission dynamics in areas where both parasites are transmitted by the same mosquito species.

## Introduction

Malaria and lymphatic filariasis (LF) are two of the most important mosquito-borne diseases. Currently, there are 2.5 billion people at risk of contracting malaria, and on average, there are 300–500 million clinical cases of malaria each year causing between one and three million deaths [1]–[3]. Human LF is caused by several species of mosquito-borne filarial nematodes, including *Brugia malayi, Brugia timori,* and *Wuchereria bancrofti*, but *W. bancrofti* is responsible for 90% of LF infections worldwide. It is estimated that 120 million people in the world have LF, with ∼1.1 billion at risk of becoming infected. Although LF is rarely fatal, severe morbidity (including adverse economic and psychosexual effects) occurs in 40% of infected individuals and involves disfigurement of the limbs and male genitalia (elephantiasis and hydrocele, respectively) [2]–[4]. Both malaria and LF are co-endemic in many areas of the tropics and in certain areas are transmitted by the same *Anopheles* mosquitoes [5]–[7]. Co-infection of multiple species of malaria parasites or a combination of malaria and filarial worm parasites in humans have been reported [7], [8], and in some cases can be quite frequent [9]. Mixed infections of these two parasites within individual mosquitoes also can occur in areas where more than one species of parasite is endemic and where *Anopheles* mosquitoes transmit both *Plasmodium* and filarial worm parasites, e.g., Papua New Guinea (PNG), rural sub-Saharan Africa, Asia, etc. (for a comprehensive review see [10]). But the interaction of co-infection between parasites and the effects on the fitness and survival of vectors is poorly known or incomplete and based only on a handful of studies (e.g., [5], [6], [8]). Moreover, traditional methods used to screen mosquitoes for the presence of parasites relied on morphological criteria for vector identification and dissection of individual mosquitoes for pathogen recovery. These approaches, however, have limitations because they are time consuming, can only provide estimates of prevalence when actual prevalence is high, do not allow the distinction of mosquito species within species complexes, and cannot differentiate species of *Plasmodium* or conclusively separate human filarial worms from those found in other animals. Recently, alternative tests have been developed for the accurate assessment of the prevalence of these pathogens in human and vector populations [10]. Therefore, an understanding of the impact mixed infections has on parasite-vector and parasite-parasite interactions are both necessary for accurate measurements of vector infection and transmission in endemic areas.

PNG is endemic for the four major species of human malaria parasites, malaria transmission occurs in all 20 PNG provinces, and malaria intensity ranges from unstable low levels of endemicity to stable malaria with year-round transmission [11]. Over one million residents of PNG are estimated to be infected with *W. bancrofti,* and the occurrence of microfilaremia, chronic disease, and acute disease is higher in PNG than in any other filariasis-endemic country [11]. Both malaria and LF are transmitted by mosquitoes in the *Anopheles punctulatus* complex in PNG [5], [11] and co-occurrence of multiple species of malaria and LF in humans is common [9] and has been shown to occur in mosquito hosts [5]. In fact, a recent study in East Sepik Province, PNG showed that 29% of individuals examined using a molecular-based assay for the simultaneous detection of the four major human *Plasmodium spp.* and *W. bancrofti* harbored both filarial worms and one or more species of malaria parasites [9]. Interactions among parasites within the same human host are known to alter disease severity [12], and the fact that two parasites might interact within the human host suggests that dual infection may alter the course of disease development and the dynamics of transmission [13], [14]. Within the vector, both *Plasmodium* and filarial worm parasites share the common developmental step of traversing the mosquito midgut, but little is known about how these two parasites interact within the vector when they share the same midgut environment. To achieve successful results in the ongoing campaigns for malaria and LF control, it is important to understand the interactions of different species of parasite with their shared hosts [3], because it has been hypothesized that eliminating *W. bancrofti* from co-endemic areas has the potential to improve the capacity of *Anopheles* to transmit *Plasmodium* [12], [15].

Accordingly, we initiated experiments to evaluate the interaction between *W. bancrofti* and *An. punctulatus* in PNG. We found that *An. punctulatus* utilizes a melanization-based immune response as a natural mechanism of resistance to filarial worms in PNG. Based on these results and the fact that *An. punctulatus* also transmits the parasites that cause malaria, we initiated laboratory experiments to test the hypothesis that immune system activation and/or development by filarial worms in mosquitoes play a role in reducing the intensity of *Plasmodium* transmission in areas where they are co-endemic. To test this hypothesis, we conducted studies using two mosquito species (*Armigeres subalbatus* and *Aedes aegypti*), three filarial worm species (*Brugia malayi, Brugia pahangi,* and *Dirofilaria immitis*), and an avian malaria parasite (*Plasmodium gallinaceum*). It was necessary to utilize model mosquito-parasite combinations to evaluate this hypothesis because there are no suitable animal models available for *W. bancrofti* and *P. falciparum*, and there is no laboratory colonized species of *Anopheles* that utilizes melanization as a natural mechanism of resistance to filarial worms. However, the melanization immune response does function as a natural mechanism of resistance to the filarial worm *B. malayi* in *Ar. subalbatus* [16], mimicking the scenario observed with *An. punctulatus* and *W. bancrofti* in PNG. Additionally, *Ar. subalbatus* used in this study are natural vectors of *P. gallinaceum* [17] and *B. pahangi* [16] and they are naturally refractory to *D. immitis*. Therefore, this unique mosquito-parasite system provides a means to assess the relationship between filarial worms and malaria parasites with the same vector in the presence or absence of an immune response or in the presence or absence of midgut penetration. The data presented herein demonstrate that when a mosquito imbibes a bloodmeal containing both malaria and filarial worm parasites, there is a significant reduction in malaria parasite development in co-infected mosquitoes regardless of whether the mosquito has an immune response to the invading filarial worms or not. However, it needs to be determined whether these results apply to *Anopheles* vectors of human malaria and lymphatic filariasis in areas of co-endemicity.

## Materials and Methods

### Ethics statement

This study was carried out in strict accordance with the recommendations in the *Guide for the Care and Use of Laboratory Animals* of the National Institutes of Health. All animals and animal facilities were under the control of the School of Veterinary Medicine with oversight from the University of Wisconsin Research Animal Resource Center and the protocol was approved by the University of Wisconsin Animal Care and Use Committee (Approval #A3368-01).

### Mosquito maintenance

*Ar. subalbatus* and *Ae. aegypti,* black-eyed Liverpool (LVP) strain, used in this study were maintained at the University of Wisconsin-Madison as previously described [18], [19]. Four- to five-day-old female mosquitoes were sucrose starved for 14 to 16 hours prior to bloodfeeding.

### Exposure to parasite-infected blood

*P. gallinaceum* infection was maintained by chicken (*Gallus gallus)* and mosquito passage. *P. gallinaceum-*infected blood was harvested from infected chickens via cardiac puncture, mixed, and split equally into two aliquots: experimental and control. *Brugia* and *D. immitis* mf were obtained from the Filariasis Research Reagent Repository Center (FR3) (Athens, Georgia, USA), filtered from cat or dog blood as described previously [20], and mixed with the experimental *P. gallinaceum-*infected blood. Microfilariae concentrations for all species of filarial worm used were approximately 50–175 mf/20 µl of blood, and *Plasmodium* gametocytemias ranged from 1–3% from biological replicate to biological replicate. Mosquitoes were exposed to bloodmeals via water-jacketed membrane feeders maintained at 36.5°C [21]. Mosquitoes that fed to repletion were separated into cartons and maintained on 0.3 M sucrose in an environmental chamber at 26.5°±1° C, 75±10% relative humidity, and with a 16 hour (h) photoperiod with a 90 minute crepuscular period. At 6 days (d) post ingestion (PI), mosquitoes were dissected, oocysts were counted, and *Plasmodium* mean intensity and prevalence was calculated. Midguts were excised in a drop of saline, transferred to a clean slide, stained with mercurochrome, and oocysts were visualized using phase contrast optics on an Olympus BH2 compound microscope at 200X magnification (Olympus America Inc., Center Valley, PA). For each biological replicate, a separate group of mosquitoes were dissected over the course of *Plasmodium* development to verify that oocysts were not being melanized. Stained and unstained midguts were examined using phase contrast optics on an Olympus Provis compound microscope at 200X and 400X magnification (Olympus America Inc., Center Valley, PA).

### Concurrent exposure to filarial worms and *P. gallinaceum*

*Ar. subalbatus* were exposed to a single bloodmeal containing a mixture of *B. malayi* mf and *P. gallinaceum* or a mixture of *B. pahangi* mf and *P. gallinaceum*. Controls were mosquitoes from the same cohort exposed to *P. gallinaceum-*infected blood. Control *P. gallinaceum-*infected blood had an equivalent amount of saline added to it to control for the saline that was added with mf to the dually infected blood even though the proportion was small. Both the concurrent ingestion of *B. malayi* mf and *P. gallinaceum* and *B. pahangi* mf and *P. gallinaceum* experiments were performed four times with separate cohorts of mosquitoes to account for stochastic variations. An additional five mosquitoes were dissected at 6 d PI to verify *B. pahangi* development and at 24 h PI to verify *B. malayi* melanization. *Ar. subalbatus* were exposed to a single bloodmeal containing a mixture of *D. immitis* mf and *P. gallinaceum*. Experimental conditions mimicked those described for concurrent ingestion of *Brugia* and *Plasmodium,* and an additional five mosquitoes were dissected at 24 h PI to confirm ingestion of *D. immitis* mf and at 6 d PI to verify that *D. immitis* was not developing. This experiment was performed three times with separate cohorts of mosquitoes.

*Ae. aegypti* were exposed to a single bloodmeal containing a mixture of *B. pahangi* mf and *P. gallinaceum.* Experimental conditions mimicked those described for concomitant infections in *Ar. subalbatus*. This experiment was performed three times. An additional two replicates were performed to assess *Plasmodium* zygote formation in *Ae. aegypti* approximately 20 h after ingestion of a co-infected bloodmeal. A single midgut was excised in a drop of saline, transferred to a clean microscope slide, homogenized in three µl of fetal bovine serum, smeared on the slide, and stained with Giemsa. Zygotes were visualized using bright field optics on an Olympus Provis compound microscope at 400X magnification (Olympus America Inc., Center Valley, PA). *P. gallinaceum* zygotes were identified as described by [17].

### Primary infection with *P. gallinaceum* followed by a secondary infection with *B. malayi*

*Ar. subalbatus* were exposed to an initial infection with *P. gallinaceum* in their first bloodmeal by feeding on ketamine/xylazine anesthetized chickens (gametocytemias =  2-4%). Six days later, following oviposition, they were exposed to a *B. malayi* infective bloodmeal in their second feeding by feeding on ketamine/xylazine anesthetized gerbils, *Meriones unguiculatus* (microfilaremias  = 100 mf/20 µl). Controls were mosquitoes from the same cohort exposed to the same *P. gallinaceum* infected chicken in their first bloodmeal and six days later, bloodfed on uninfected gerbils. This experiment was repeated (n =  2 biological replicates) with separate cohorts of mosquitoes. At 2 days following their second feeding (8 days post *P. gallinaceum* exposure), mosquitoes were dissected to determine *Plasmodium* mean intensity and prevalence.

### Concurrent exposure to *P. gallinaceum* plus *B. malayi* excretory/secretory products

*B. malayi* microfilariae (50 mf/20 µl) were cultured in serum-free RPMI 1640 (GIBCO) supplemented with five g/L glucose and antibiotic-antimycotic (Invitrogen, 100 U/ml penicillin, 100 µg/ml streptomycin, and 0.25 µg/mL amphotericin B). Spent media was collected and replaced with fresh media every 24 hours to a maximum time of 3 days. The medium collected was filtered through 0.2 µM filters (Millipore), pooled and concentrated using Amicon Ultrafilters with 3 kDa cut-off membranes, and stored at −80°C until use. Excretory/secretory (E/S) product concentrations were estimated based on OD_280_ using a Nanodrop ND-1000 Spectrophotometer (Thermo Fisher Scientific, San Jose, CA) [22]. *P. gallinaceum-*infected blood was supplemented with B. *malayi* E/S product to a final concentration of 0.025 mg/ml and fed to mosquitoes via a water-jacketed membrane feeder. Controls were mosquitoes from the same cohort exposed to the same *P. gallinaceum-*infected blood supplemented with an equivalent amount of media added as was added with E/S product. At 6 d PI, 50 mosquitoes were dissected to determine *Plasmodium* mean intensity and prevalence.

### Inoculation of *D. immitis* microfilariae into *P. gallinaceum* exposed mosquitoes

*Ar. subalbatus* were initially exposed to *P. gallinaceum* by feeding on a ketamine/xylazine anesthetized chicken, with a gametocytemia of 3.2%. Immediately following exposure to *P. gallinaceum*, fully blood fed mosquitoes were intrathoracically injected with approximately 200 *D. immitis* mf. Controls were mosquitoes from the same cohort exposed to the same *P. gallinaceum-*infected chicken. Immediately, following blood feeding, approximately 0.5 µl of *Aedes* saline, without mf, was intrathoracically injected into mosquitoes of the control group. At 6 d PI, 38 mosquitoes were dissected to determine oocyst mean intensity in the control group and 21 mosquitoes in the experimental group. An additional five mosquitoes were dissected at 24 h PI to verify *D. immitis* melanization.

### Statistical analysis

Mean intensity is here defined as the mean number of oocysts per infected mosquito. Prevalence is defined as the number of infected hosts per the number of hosts examined. Comparisons of prevalence were analyzed using an Exact unconditional test, and comparisons of mean intensity were analyzed using a Bootstrap *t*-test as described in [23] and [24]. Statistical tests were run using Quantitative Parasitology 3.0, a software package designed to analyze the highly aggregated frequency distributions exhibited by parasites [23].

## Results and Discussion

### The interaction of *W. bancrofti* with *An. punctulatus* in PNG

In PNG, the interaction of *W. bancrofti* with its *Anopheles* vectors has generally been considered one of facilitation, i.e., the proportional conversion of infective-stage larvae (L3) in mosquito vectors increases as the density of circulating microfilariae (mf) increases from very low numbers (e.g., ∼10 mf/ml blood) to intermediate levels (e.g., ∼100 mf/ml blood). When mf densities are relatively high (e.g., ∼1000 mf/ml blood), however, there is a reduction in the intensity of mosquito infections [25]. It has been reported previously that prevalence of *W. bancrofti* ranged from 2% to 11.7% in *An. punctulatus* in East Sepik Province [26], and sporozoite rates of the *An. punctulatus* group of mosquitoes seldom exceed 3% [27]. We conducted experiments at the field station facilities of the PNG Institute of Medical Research in Maprik to evaluate the interaction between *W. bancrofti* and *An. punctulatus*. Mosquitoes (n = 418) were collected in the village complex of Drekikire in the early morning as they rested inside village homes. Seventy-two of the dissected mosquitoes harbored some stage of *W. bancrofti* (17.2% prevalence) and a total of 242 parasites were recovered (101 mf, 71 L1, 56 L2, and 14 L3). Of the 72 infected mosquitoes, nearly 50% (35/72) employed an innate immune response called melanization against these parasites (see [28]), and a total of 54 parasites were melanized and killed. In addition, 14 of the infected mosquitoes had killed all of their parasites, providing an estimated resistance rate of 19.4%. This is one of the few instances where melanization has been shown to function as a primary mechanism controlling resistance in a natural vector population (see [28]), and it seems that this response is the primary factor controlling facilitation in this mosquito-parasite interaction. Based on these data and previous reports, we hypothesized that immune system activation and/or development by filarial worms in mosquitoes play a role in reducing the intensity of *Plasmodium* transmission in areas where they are co-endemic [12], [15].

### Exposure to filarial worms influences the mosquitoes' permissiveness to *Plasmodium* infection

When *Ar. subalbatus* ingests mf in a bloodmeal, penetration of the mosquito midgut epithelium occurs shortly after ingestion (within minutes) [29]. If *Ar. subalbatus* ingests mf of *B. pahangi*, migration to the thoracic musculature follows and is complete by approximately 12 h PI. If the mosquito ingests mf of *B. malayi,* midgut penetration occurs, but mf are rapidly melanized in the hemocoel [30], [31]. At 24 to 48 h PI mf begin to die, and by 72 h PI, the response is all but complete [18], [32]. In contrast, *P. gallinaceum* penetration into the mosquito midgut is comparatively a much longer process than filarial worm penetration, i.e., filarial worms penetrate in a matter of minutes whereas malaria parasites penetrate many hours after ingestion. Ingestion of *P. gallinaceum* gametocytes by *Ar. subalbatus* during a bloodmeal activates the formation of gametes in the mosquito midgut lumen, which undergo syngamy to form a zygote. The zygotes transform into motile ookinetes 30 h later, move out of the blood bolus, and migrate across the peritrophic matrix [17], [33]–[35]. Ookinetes exit the midgut epithelium through the basal end and transform into sessile oocysts, which are evident on the midgut approximately 2 d PI. Therefore, *P. gallinaceum* development was assessed 6 d PI, i.e., a time when development was well established and easily visualized.

**Figure 1:**
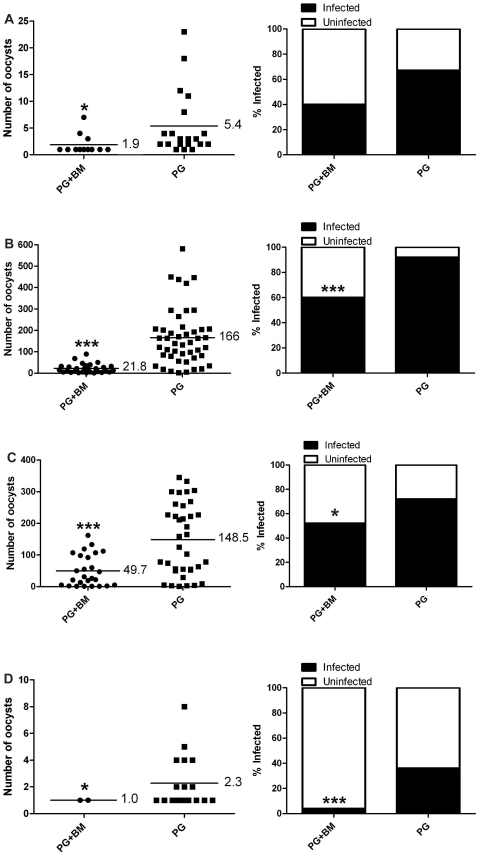
*Plasmodium* infection in *Ar. subalbatus* that concurrently ingested *P. gallinaceum* and *B. malayi.* Mosquitoes that fed on blood containing *P. gallinaceum* alone served as controls. For all, the left panel indicates infection intensity where points indicate the absolute value of oocyst counts in individual mosquitoes, and horizontal black bars represent the mean intensity. The right panel indicates prevalence of infection where the bars represent the total population of mosquitoes examined. The filled portion of the bars indicates the proportion of mosquito midguts that were positive for at least one oocyst; the unfilled portion of the bar indicates the proportion of midguts that were uninfected. PG, *P. gallinaceum*; BM, *B. malayi;* *, significant reduction in mean intensity and prevalence (*p<0.05, **p<0.01, ***p<0.001). **A**.) Biological replicate number 1, n = 30. **B**.) Biological replicate number 2, n = 50. **C**.) Biological replicate number 3, n = 50. **D**.) Biological replicate number 4, n = 50.

Our first goal was to determine if a melanization-based immune response activated by *B. malayi* had any effect on *P. gallinaceum* development in *Ar. subalbatus*. Mosquitoes that ingested blood containing *P. gallinaceum* alone (control) or both *P. gallinaceum* and *B. malayi* (experimental) were assessed for *Plasmodium* development, and there was a significant reduction in the intensity (Bootstrap *t-*test) of *Plasmodium* infection in mosquitoes exposed to both parasites as compared to *P. gallinaceum* alone (Figure 1A–D), and there was no evidence of melanization against oocysts in any replicate. In three of the replicates, there also was a significant reduction in the prevalence (Exact unconditional test) of infection (Figure 1B–D) and for the other there was a close to statistically significant reduction (*p = *0.052) (Figure 1A). These results supported our initial hypothesis that immune system activation by filarial worms in mosquitoes negatively affects *Plasmodium* development, but it was not clear if activation of the mosquito's immune system by filarial worms was in fact mediating the reduction and not some other phenomenon.

**Figure 2:**
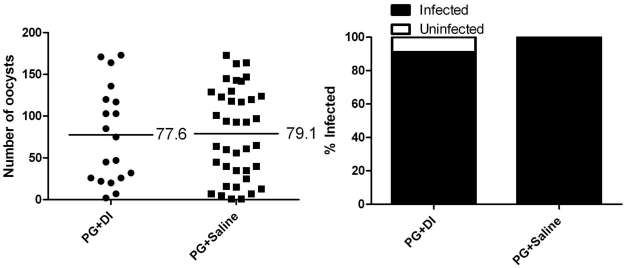
*Plasmodium* infection in mosquitoes inoculated with *D. immitis* mf immediately following blood feeding. Mosquitoes that fed on blood containing *P. gallinaceum* and received a saline inoculation immediately following blood feeding served as controls. There was no significant difference in *Plasmodium* development between mosquitoes that were exposed to *P. gallinaceum* and intrathoracic injection of saline (n = 38) or mosquitoes exposed to *P. gallinaceum* and intrathoracic injection of *D. immitis* mf (n = 21). The left panel indicates infection intensity where points indicate the absolute value of oocyst counts in individual mosquitoes, and horizontal black bars represent the mean intensity. The right panel indicates prevalence of infection where the bars represent the total population of mosquitoes examined. The filled portion of the bars indicates the proportion of mosquito midguts that were positive for at least one oocyst; the unfilled portion of the bar indicates the proportion of midguts that were uninfected. PG, *P. gallinaceum;* DI, *D. immitis.*

**Figure 3:**
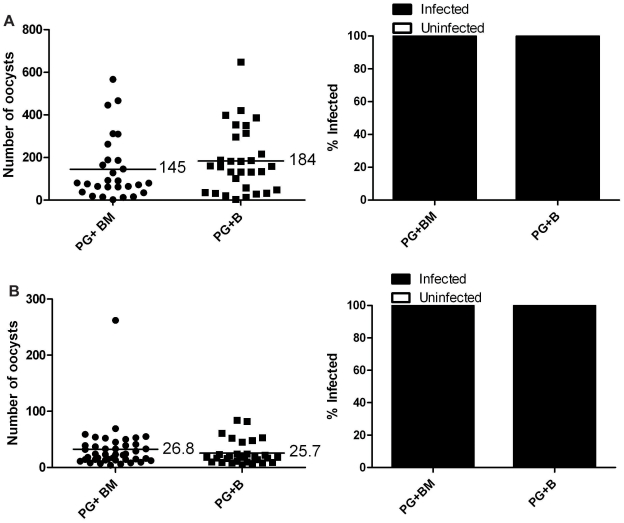
A secondary exposure to *B. malayi* does not affect *Plasmodium* development. Mosquitoes that had a primary exposure to *P. gallinaceum* and a secondary exposure to uninfected blood served as controls. Mosquitoes that had a primary exposure to *P. gallinaceum* and a secondary exposure to *B. malayi* served as the experimental group. For both, the left panel indicates infection intensity where points indicate the absolute value of oocyst counts in individual mosquitoes, and horizontal black bars represent the mean intensity. The right panel indicates prevalence of infection where the bars represent the total population of mosquitoes examined. The filled portion of the bars indicates the proportion of mosquito midguts that were positive for at least one oocyst; the unfilled portion of the bar indicates the proportion of midguts that were uninfected. There was no significant difference in *Plasmodium* development between mosquitoes that received a primary exposure to *P. gallinaceum* followed by a secondary exposure to uninfected blood or mosquitoes that received a primary exposure to *P. gallinaceum* followed by a secondary exposure to *B. malayi-*infected blood. PG, *P. gallinaceum*; BM, *B. malayi*; B, uninfected blood. **A**.) Biological replicate number 1, PG+BM n = 28, PG+B n = 30. **B**.) Biological replicate number 2, PG+BM n = 42, PB+B n = 30.

To ascertain if the melanization immune response was mediating the reduction in *Plasmodium* development, we activated this immune response in the absence of midgut penetration by filarial worms. Melanization was activated by intrathoracic inoculation of *D. immitis* mf, which stimulates an extremely robust melanization immune response in the hemocoel of *Ar. subalbatus* [36]. Mosquitoes that ingested blood containing *P. gallinaceum* plus an intrathoracic inoculation of *D. immitis* mf were assessed for *Plasmodium* development and compared with control mosquitoes inoculated with saline without mf. Microscopic examination of midguts from each group indicated no difference in the intensity or prevalence of *Plasmodium* infection (Figure 2). We then conducted experiments to test if melanization had a negative effect on an established *P. gallinaceum* infection. Mosquitoes with an established *P. gallinaceum* infection were exposed to a subsequent bloodmeal containing *B. malayi* mf or an uninfected bloodmeal 6 d following the initial exposure to a bloodmeal containing *P. gallinaceum* gametocytes. Mosquito midguts were analyzed 48 h after the subsequent bloodmeal and there was no difference in the intensity or prevalence of *Plasmodium* infection (Figure 3A and B). These results suggested that filarial worm activation of a melanization immune response was not mediating the reduction in *Plasmodium* development.

### Filarial worm penetration through the mosquito midgut affects *Plasmodium* development

**Figure 4:**
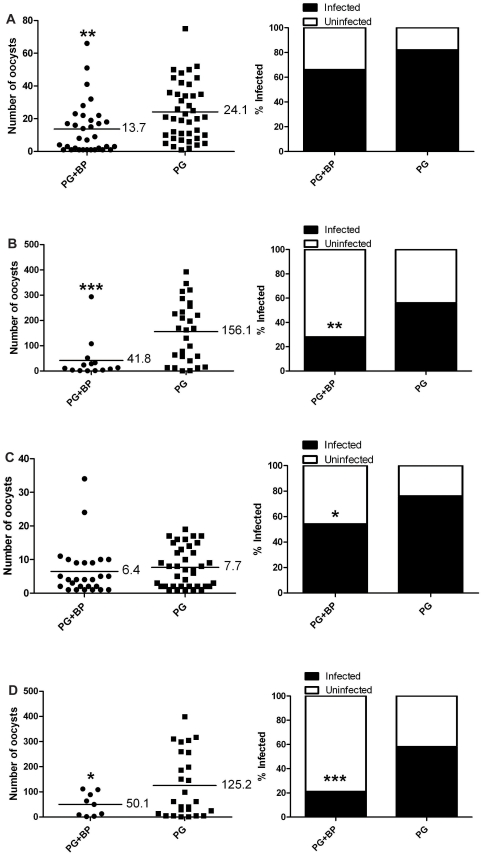
*Plasmodium* infection in *Ar. subalbatus* that concurrently ingested *P. gallinaceum* and *B. pahangi.* Mosquitoes that fed on blood containing *P. gallinaceum* alone served as controls. For all, the left panel indicates infection intensity where points indicate the absolute value of oocyst counts in individual mosquitoes, and horizontal black bars represent the mean intensity. The right panel indicates prevalence of infection where the bars represent the total population of mosquitoes examined. The filled portion of the bars indicates the proportion of mosquito midguts that were positive for at least one oocyst; the unfilled portion of the bar indicates the proportion of midguts that were uninfected. PG, *P. gallinaceum*; BP, *B. pahangi;* *, significant reduction in mean intensity and prevalence (*p<0.05, **p<0.01, ***p<0.001). **A**.) Biological replicate number 1, n = 50. **B**.) Biological replicate number 2, n = 50. **C**.) Biological replicate number 3, n = 50. **D**.) Biological replicate number 4, PG+BP n = 43, PG+B n = 45.

We then investigated whether or not filarial worm development (in the absence of melanization) had a negative effect on *P. gallinaceum* development in *Ar. subalbatus*. Mosquitoes that fed on blood containing *P. gallinaceum* alone or containing both *P. gallinaceum* and *B. pahangi* (*Ar. subalbatus* supports the complete development of *B. pahangi*) were assessed for *Plasmodium* development, and there was a significant reduction in the intensity (Figure 4A, B, and D) and prevalence (Figure 4B–D) of *Plasmodium* infection. In one group (Figure 4C) there was no difference in intensity; however, within this group there were two mosquitoes with dual infections that harbored 33% of the total oocysts recovered, and if these two mosquitoes are removed from the data set, there is a significant reduction in the intensity of *Plasmodium* infection (Figure 4C). These results suggested that midgut penetration, regardless of the melanization-based immune response, was mediating the reduction in *Plasmodium* development in co-infected mosquitoes; therefore, we postulated that the reduced infectivity of mosquitoes for *P. gallinaceum* is directly, or indirectly, related to filarial worm penetration.

**Figure 5:**
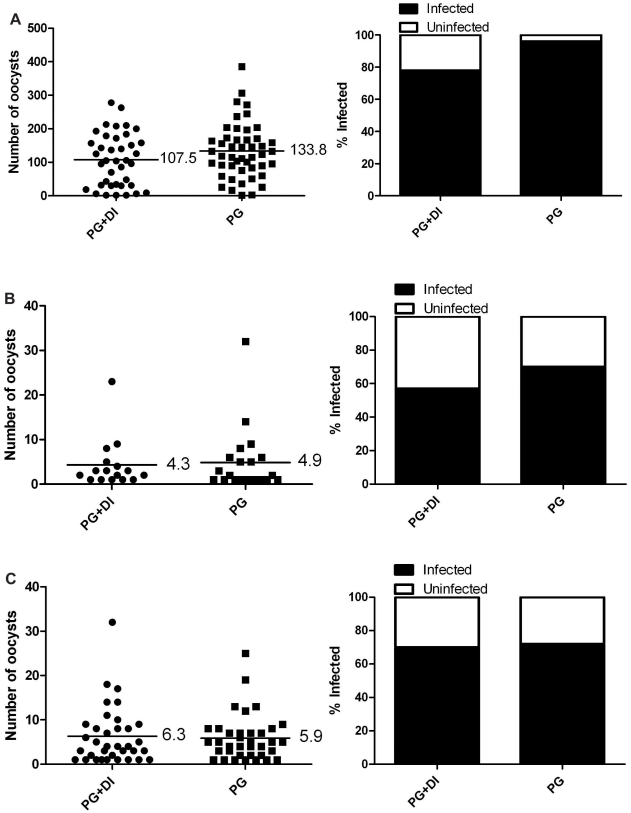
*Plasmodium* infection in *Ar. subalbatus* that concurrently ingested *P. gallinaceum* and *D. immitis.* Mosquitoes that fed on blood containing *P. gallinaceum* alone served as controls. For all, the left panel indicates infection intensity where points indicate the absolute value of oocyst counts in individual mosquitoes, and horizontal black bars represent the mean intensity. The right panel indicates prevalence of infection where the bars represent the total population of mosquitoes examined. The filled portion of the bars indicates the proportion of mosquito midguts that were positive for at least one oocyst; the unfilled portion of the bar indicates the proportion of midguts that were uninfected. There was no significant difference between mosquitoes that were exposed to *P. gallinaceum* alone or mosquitoes exposed to a bloodmeal that contained both *P. gallinaceum* and *D. immitis.* PG, *P. gallinaceum;* DI, *D. immitis.* **A**.) Biological replicate number 1, PG+DI n = 50, PG+B n = 49. **B**.) Biological replicate number 2, PG+DI n = 28, PG+B n = 30. **C**.) Biological replicate number 3, n = 50.

**Figure 6:**
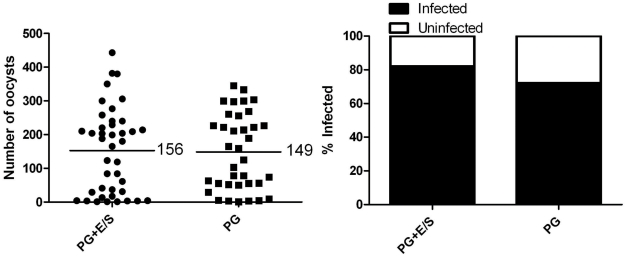
*B. malayi* E/S products have no effect on *Plasmodium* infection. *Plasmodium* infection intensity and prevalence in *Ar. subalbatus* exposed to *P. gallinaceum-*infected blood supplemented with *B. malayi* E/S products. Mosquitoes that fed on blood containing *P. gallinaceum* alone served as controls. Points indicate the absolute value of oocyst counts in individual mosquitoes, and horizontal black bars represent the mean intensity. There was no significant difference in *Plasmodium* development between mosquitoes that were exposed to *P. gallinaceum* alone (n = 50) or mosquitoes exposed to a bloodmeal that contained *P. gallinaceum* supplemented with *B. malayi* E/S products (n = 50). The left panel indicates infection intensity where points indicate the absolute value of oocyst counts in individual mosquitoes, and horizontal black bars represent the mean intensity. The right panel indicates prevalence of infection where the bars represent the total population of mosquitoes examined. The filled portion of the bars indicates the proportion of mosquito midguts that were positive for at least one oocyst; the unfilled portion of the bar indicates the proportion of midguts that were uninfected. PG, *P. gallinaceum*; E/S, *B. malayi* excretory/secretory products.

Our next goal was to verify that midgut penetration by mf was contributing to the reduction in *P. gallinaceum* development in co-infected *Ar. subalbatus.* Mosquitoes that fed on blood containing *P. gallinaceum* alone or containing both *P. gallinaceum* and *D. immitis* (mf present in the bloodmeal but no midgut penetration) were assessed for *Plasmodium* development. *D. immitis* is a filarial worm that does not penetrate the midgut of mosquitoes, rather it develops in the Malpighian tubules. In *Ar. subalbatus, D. immitis* travels to the Malpighian tubules but does not develop past the mf stage. This failure to develop is probably due to a physiological incompatibility and seems to be independent of an active immune response [37]. There was no difference in *Plasmodium* development in mosquitoes exposed to both parasites (Figure 5A–C) as compared to *P. gallinaceum* alone. Additionally, filarial worm excretory/secretory (E/S) products released in the mosquito midgut were not found to reduce *P. gallinaceum* development in mosquitoes that ingested *P. gallinaceum*-infected blood supplemented with *B. malayi* E/S products as compared to mosquitoes that fed on blood infected with *P. gallinaceum* alone (Figure 6). These results strongly suggest that midgut penetration by filarial worms is directly, or indirectly, responsible for a reduction in *Plasmodium* development in co-infected mosquitoes.

### The anti-*Plasmodium* effect of microfilariae can be repeated in another mosquito species

**Figure 7:**
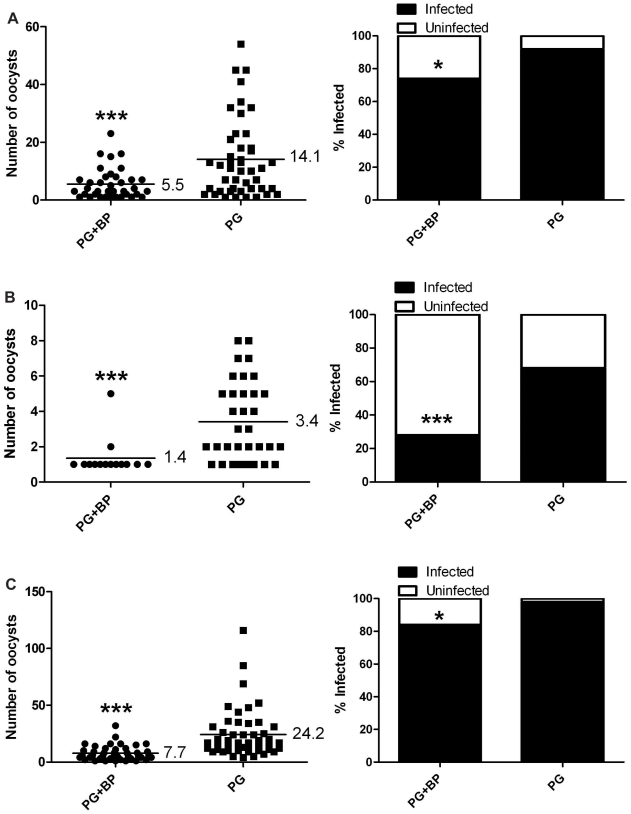
*Plasmodium* infection in *Ae. aegypti* that concurrently ingested *P. gallinaceum* and *B. pahangi.* Mosquitoes that fed on blood containing *P. gallinaceum* alone served as controls. For all, the left panel indicates infection intensity where points indicate the absolute value of oocyst counts in individual mosquitoes, and horizontal black bars represent the mean intensity. The right panel indicates prevalence of infection where the bars represent the total population of mosquitoes examined. The filled portion of the bars indicates the proportion of mosquito midguts that were positive for at least one oocyst; the unfilled portion of the bar indicates the proportion of midguts that were uninfected. PG, *P. gallinaceum*; BP, *B. pahangi;* *, significant reduction in mean intensity and prevalence (*p<0.05, **p<0.01, ***p<0.001). **A**.) Biological replicate number 1, n = 50. **B**.) Biological replicate number 2, n = 50. **C**.) Biological replicate number 3, n = 50.

Finally, we tested if the reduction in *P. gallinaceum* development was mediated by the specific physiology of the *Ar. subalbatus* midgut or if this phenomenon could be repeated in another species of mosquito using the same parasites. *Ae. aegypti,* black-eyed Liverpool strain (which supports the complete development of *B. pahangi* and *P. gallinaceum*), that fed on blood containing both *B. pahangi* and *P. gallinaceum* or *P. gallinaceum* alone were assessed for *Plasmodium* development, and there was a significant reduction in the intensity and the prevalence of *Plasmodium* infection (Figure 7A–C) in co-infected mosquitoes 6 d post bloodfeeding. Additionally, no observed difference in the intensity or the prevalence of *Plasmodium* infection or in zygote morphology at 20 h post infection in the same mosquitoes suggested that the presence of mf does not affect *Plasmodium* syngamy or zygote formation in co-infected bloodmeals. These results also demonstrate that the reduced infectivity of *P. gallinaceum* in the presence of filarial worms could be repeated in another mosquito species.

In sum, concurrent ingestion of *Brugia* mf and *P. gallinaceum* gametocytes significantly affects the development of *P. gallinaceum* in co-infected mosquitoes. This was demonstrated by a significant reduction in both malaria parasite intensity and prevalence in *Ar. subalbatus* mosquitoes with double infections and is independent of whether the mosquito has an immune response to the filarial worm (*B. malayi*) or not (*B. pahangi*). These results lead to our belief that the reduction is related (either directly or indirectly) to microfilarial penetration through the mosquito midgut. Consistent with this belief is the fact that we did not observe a significant effect on *Plasmodium* development in mosquitoes that concurrently ingested *P. gallinaceum* gametocytes and *D. immitis* mf, *P. gallinaceum* gametocytes and *B. malayi* E/S products, *P. gallinaceum* gametocytes followed by intrathoracic inoculation of *D. immitis* mf (melanization activated but no midgut penetration), or no effect on zygote formation in *Ae. aegypti* that ingested *P. gallinaceum* gametocytes and *B. pahangi* mf. In addition, Albuquerque and Ham (1995) showed no difference in oocyst numbers (using their untransformed data) in *Plasmodium*-infected *Ae. aegypti* when *B. pahangi* mf were inoculated into the hemocoel at 4 d post *P. gallinaceum* infection [38], thereby enabling filarial worm development or immune activation without midgut penetration by mf.

There are several mechanisms associated with midgut penetration by filarial worms that could account for this reduction in *Plasmodium* infectivity. One possibility is that damage to midgut tissue could interfere with the ability of ookinetes to traverse the midgut epithelium.

In *Ae. aegypti,* when *Brugia* mf penetrate the midgut, pathology extends across two to four adjacent cells (e.g., the cytoplasm of adjacent cells contains vacuolated mitochondria and pycnotic nuclei) surrounding the point of penetration and disrupts the full depth of the midgut wall resulting in the destruction of cellular integrity (i.e., the basal plasma membrane is disrupted and the underlying musculature is torn and partially dislodged) [39], and this could result in the destruction of the intracellular junctions necessary for ookinete entry into midgut cells [40]–[42]. Similar pathological consequences have been observed in *An. gambiae* and *Ae. aegypti* following *W. bancrofti* infection, i.e., microfilarial penetration caused the cytoplasm of affected cells to become basophilic and their nuclei to become pycnotic [43]. The only major difference was that *W. bancrofti-*infected *An. gambiae* midgut cells showed evidence of hypertrophy, a phenomenon that has not been observed in *Ae. aegypti* infected with either *Brugia* or *W. bancrofti* [39], [43]. And it has been shown that pathology associated with *P. gallinaceum* invasion into *Ae. aegypti* midgut cells persists for at least 24 h post infection [44]; therefore, the pathology associated with filarial worm penetration persists for a period of time that is long enough to have an influence on ookinete migration out of the midgut. The suggestion that midgut damage might interfere with ookinete migration through the midgut also was proposed by Kala and Gunasekaran (1999), in studies where *Ae. aegypti* co-infected with *P. gallinaceum* and *Bacillus thuringiensis ssp. israelensis* (*Bti*) had a significant reduction in *Plasmodium* development as compared to controls. These authors suggested that the *Bti* toxin disrupted the midgut epithelium and interfered with the ability of ookinetes to invade midgut epithelial cells [45].

A second mechanism is that midgut penetration by filarial worms activates alternative immune-mediated mechanisms against invading mf- even if the mosquito supports the development of filarial worms- that are also active against *Plasmodium* parasites (e.g., reactive intermediates of nitrogen and oxygen, antimicrobial peptides, etc.). Both *Ar. subalbatus* and *Ae. aegypti* support the development of *B. pahangi,* but parasite tolerance may involve immunological mechanisms directed at tissue damage or other harmful substances resulting from infection with filarial worms, or may even reflect the filarial worm's ability to persistently evade the host's defenses to remain inside the host to achieve eventual transmission [46]. A number of transcripts implicated in innate immunity showed significantly different transcriptional behavior as a result of *B. pahangi* infection vs. uninfected blood in a study previously conducted by our laboratory [31], and similar results were shown in a study examining the infection response of *Ae. aegypti* to *B. malayi* [47]. And these immune mechanisms could be detrimental to *Plasmodium* development (especially considering that their induction loosely coincides with the time *Plasmodium* parasites are most vulnerable) in concomitantly infected mosquitoes, i.e., a particular gene may be involved in both tolerance and resistance to filarial worms but also may an have anti-*Plasmodium* effect, because resistance and tolerance can be mutually exclusive, interchangeable, or complementary components of a mixed strategy of defense [48] depending on the pathogens involved.

A third possible mechanism involves the physical disruption of the midgut that could facilitate leakage of mosquito midgut bacteria into the hemocoel in a manner similar to what has been observed with concomitant infection involving filarial nematodes and arbovirus, i.e., physical disruption of the midgut facilitates virus penetration into the hemocoel and enhances the vector's susceptibility to the arbovirus. In contrast, bacterial leakage into the hemocoel could be inducing a suite of antimicrobial factors that also are detrimental to *Plasmodium* development [49].

An additional mechanism could be related to mf-induced pathology and the subsequent repair of the midgut having a detrimental effect on *Plasmodium* development. In our laboratory's previous transcriptomic analyses of filarial worm associated gene expression, a number of transcripts previously implicated in apoptosis showed significantly different transcriptional behavior (e.g., cathepsin, calcium-independent phospholipase, etc.) [30], [31], and cell death in vertebrates has been shown to trigger both innate and adaptive immune responses [50], [51]. The destruction of basal and apical plasma membranes by penetrating mf likely results in cell death [39] and the resultant restitution of the midgut could negatively impact *Plasmodium* development. In *Drosophila,* midgut homeostasis is maintained following pathogenic bacterial infection or physical stress via the induction of cytokines in the *Unpaired* family. In intestinal stem cells, these cytokines activate the Jak/Stat signaling pathway, which promotes proliferation of intestinal stem cells, and also causes a gut-specific immune response in enterocytes, leading to the production of antimicrobial peptides [52]. Although there is no experimental evidence to support this phenomenon in mosquitoes, similar processes do occur (e.g., [53]), and there are transcriptomic data from our previous studies implicating the possible involvement of a number of different signal transduction molecules, cell cycle regulators, and antimicrobial peptides in both susceptibility and refractoriness of filarial worms to mosquitoes [30], [31] that could be having inadvertent negative consequences on *Plasmodium* development.

Regardless of the mechanism involved in mediating this phenomenon, if this laboratory model of concomitant infection is representative of what occurs naturally in areas where both malaria parasites and filarial worms are transmitted to humans by the same *Anopheles* vector, then the possibility exists that the elimination of filarial worms in a co-endemic locale could enhance malaria transmission. A study conducted on the Kenyan coast by Muturi et al. (2006) came to a similar conclusion even though their results showed higher sporozoites rates in *W. bancrofti-* and *P. falciparum-*infected *An. gambiae.* They suggested that enhancement of malaria transmission also could occur as a result of the reduction in filarial worm-induced mosquito mortality in co-endemic areas following elimination of LF [8]. It is important to consider that transmission intensity is a function of both the prevalence and intensity of infection. And our results showed a statistically significant decrease in both measures of infection in co-infected mosquitoes, which further supports the possibility of inadvertent enhancement of malaria with the elimination of LF. Additionally, the evidence provided here is consistent with studies that have examined malaria and LF co-infection in mosquitoes (for review see [3], [10]), i.e., despite the two parasites sharing common vectors and environmental factors necessary for development, one parasite tends to dominate the other in a mixed infection [5], [6], [8], [12].

At the very least, these results warrant further exploration, both in the laboratory and in the field, of the interaction of human malaria and filarial worm parasites when they co-infect an *Anopheles* species that functions as a natural vector for both parasites. Such studies would help to determine if attempts to control one parasite may inadvertently lead to a change in prevalence of the other [12]; because, the control of either disease depends on sufficient epidemiological knowledge before being able to propose and implement a sound intervention strategy [10]. This becomes increasingly important considering that the main aim of the Global Programme to Eliminate Lymphatic Filariasis (GPELF) is to achieve worldwide elimination of the parasites that cause this disease through mass drug administration by the year 2020 [54]. More importantly, the evidence provided here supports the argument for the expansion of vector control based on integrated control strategies targeting both LF and malaria [55]–[57]. Resources can be limited in many countries endemic for malaria and LF; therefore, integrating control efforts for these two diseases should be a priority. Integrated vector control has been extremely successful in the past (e.g., PNG, Kenya, and the Solomon Islands), and it is the most cost-effective approach to achieving simultaneous malaria and LF reduction or outright elimination [10]. In fact, in many situations, the timeline of the GPELF might be achieved more rapidly by incorporating vector control strategies into their program [58].
